# Mechanism of kinetin-induced death of *Vicia faba* ssp. *minor* root cortex cells

**DOI:** 10.1038/s41598-021-03103-3

**Published:** 2021-12-09

**Authors:** Andrzej Kaźmierczak, Anita Kunikowska, Magdalena Doniak, Andrzej Kornaś

**Affiliations:** 1 Department of Cytophysiology, Institute of Experimental Biology, Faculty of Biology and Environmental Protection, University of Łódź, Pomorska 141/143, 90-236 Lodz, Poland; 2 Institute of Biology, Pedagogical University of Krakow, Podchorążych 2, 30-084 Kraków, Poland

**Keywords:** Cell death, Root apical meristem, Kinases, Plant cell death

## Abstract

Cell death (CD) may be induced by endogenous or exogenous factors and contributes to all the steps of plant development. This paper presents results related to the mechanism of CD regulation induced by kinetin (Kin) in the root cortex of *Vicia faba* ssp. *minor*. To explain the process, 6-(2-hydroxy-3-methylbenzylamino)purine (PI-55), adenine (Ad), 5′-amine-5′-deoxyadenosine (Ado) and N-(2-chloro-4-piridylo)-N′-phenylurea (CPPU) were applied to (i) block cytokinin receptors (CKs) and inhibit the activities of enzymes of CK metabolism, i.e., (ii) phosphoribosyltransferase, (iii) kinases, and (iv) oxidases, respectively. Moreover, ethylene glycol-bis(*β*-aminoethyl ether)-N,N,N′,N′-tetraacetic acid (EGTA), lanthanum chloride (LaCl_3_), ruthenium red (RRed) and cyclosporine A (CS-A) were applied to (i) chelate extracellular calcium ions (Ca^2+^) as well as blocks of (ii) plasma-, (iii) endoplasmic reticulum- (ER) membrane Ca^2+^ ion channels and (iv) mitochondria- (MIT) Ca^2+^ ions release by permeability transition por (PTP), respectively. The measured physiological effectiveness of these factors was the number of living and dying cortex cells estimated with orange acridine (OA) and ethidium bromide (EB), the amounts of cytosolic Ca^2+^ ions with chlortetracycline (CTC) staining and the intensity of chromatin and Ca^2+^-CTC complex fluorescence, respectively. Moreover, the role of sorafenib, an inhibitor of RAF kinase, on the vitality of cortex cells and ethylene levels as well as the activities of RAF-like kinase and MEK2 with Syntide-2 and Mek2 as substrates were studied. The results clarified the previously presented suggestion that Kin is converted to appropriate ribotides (5′-monophosphate ribonucleotides), which cooperate with the ethylene and Ca^2+^ ion signalling pathways to transduce the signal of kinetin-programmed cell death (Kin-PCD). Based on the present and previously published results related to Kin-PCD, the crosstalk between ethylene and MAP kinase signalling, as well as inhibitors of CK receptors and enzymes of their metabolism, is proposed.

## Introduction

Programmed cell death (PCD) is a developmentally and environmentally (biotically or abiotically) induced process in all organisms^1,2^.

The ontogenesis of all steps of plant growth and development, from their reproduction to senescence^1–3^, is controlled by developmental PCD (dPCD)^1–3^, while an environmentally (biotically or abiotically) induced process is called environmental PCD (ePCD)^1–3^. dPCD is related to modification of cell functions or cell elimination and control of the formation of vegetative (e.g., xylem and phloem tissues) and generative (e.g., embryo)^1–3^ organs. PCD can be induced by exogenously applied plant hormones, one of which, in addition to ethylene (ETH)^4^ or 1-aminocyclopropane-1-carboxylic acid (ACC), is kinetin (Kin)^5,6^, which induces the death of root cortex cells of *Vicia faba* spp. *minor* seedlings^7–11^.

Hallmarks of kinetin-programmed cell death (Kin-PCD)^6–11^ have been extensively and thoroughly^12^ scrutinized, showing that Kin induces the degradation of approximately 50% of cortex cells, making cell-free spaces called aerenchyma^6,9^. Aerenchyma is an important tissue that allows plants to exchange respiratory gases (oxygen and carbon dioxide) between roots and shoots in aquatic and wetland environments^4^. The most common morphologic features of Kin-PCD consist of degradation of some or all cellular compartments^6,9^, in which nuclei^7,8^ and/or cell walls^6^ are degraded last. The progression of this process is directly related to ACC because its application^13^ confirmed such divagation.

Kin induces the elimination of cortex cells but not seedlings^9^. The results indicate that this developmental regulator plays a dual role, i.e., it triggers disruption of some of the destined-to-die cells and simultaneously prevents death in others^11^. The latter was evidenced by greater activities of enzymes involved in reactive oxygen species (ROS) scavenging (catalases and superoxide dismutase)^10^ and by elevated amounts of cellulose, callose and other cell wall-bound sugars, leading to thickening of the walls of living cells not destined for death. Moreover, transmission electron microscopy showed a lack of plasmodesma connections between living and dying cortex cells^11^. This is in accordance with data indicating that cytokinins (CKs) are transported by symplasmic connections between cells within plants^11,14^.

Kin-PCD is also hallmarked by: (i) a lower number of mitochondria (MIT) and their malformed morphology induced by ROS overproduction^11^; (ii) nuclear chromatin condensation and chromatin fragmentation with exo-/endonucleolytic enzymes^7,8^; (iii) the appearance of enormous acidic lytic vacuoles^9^; (iv) unchanging amounts of proteins^10^; (v) fluctuation of serine- and cysteine-dependent protease activities^10^; (vi) a decrease in the number of dying cortex cells after treatment with N-ethylmaleimide (NEM), phenylmethylsulfonyl fluoride (PMSF) and Z-Leu-Leu-Nva-H (MG115), inhibiting the influence of MG115 on the proteolytic activities of *β*1 proteasome subunits that play an important role in the signal transduction pathway during Kin-PCD, mimicking caspase-like signalling elements^10^.

Moreover, during Kin-PCD, similar to apoptosis in *Caenorhabditis elegans*^15^, the specification, executive and degradation phases^8,10^ were distinguished for the first time for plant cell death. The phases may last from 0 to 3 h, from 6 to 18 h and from 24 to 96 h, respectively^8,10^. In the specification (signalling) phase, the total and cytosolic levels of calcium ions (Ca^2+^) were highest in the cortex of apical parts of faba bean seedling roots and cortex cells, respectively, as well as the amount of ACC in these fragments^6^. The ROS levels were highest in the executive phase, and the activity of ROS metabolism enzymes and the amount of free sugars were highest in the degradation phase^6^.

Considering all the hallmarks that were studied in relation to Kin-PCD allowed us to suggest that Kin induces vacuolar^9^ or autolytic^7^ types of cell death. This suggestion is reflected in Locato and De Gara^2^ paper.

The studies whose results are presented in the paper were undertaken to prove the hypothesis presented in Kunikowska et al*.*^9^, which suggested that Kin induces cell death (CD) after its conversion with phosphoribosyl transferase to corresponding monophosphates, which are purine-specific ligands^16–18^ for one or two histidine kinase (HK) CK receptors^18,19^. This hypothesis is based on data showing that free CK bases exogenously applied into organisms are rapidly converted into their nucleosides and/or nucleotides^16–18^.

Moreover, treatment of *Arabidopsis thaliana* cells with benzyl adenine (BA) induced CD only in the presence of HK4 receptors^20^, although it had low affinity for BA^19^. In *A. thaliana,* HK3 had an approximately tenfold lower affinity for isopentenyladenine and its riboside but a higher affinity than HK4 for dihydrozeatin and zeatin and isopentenyl adenine/cytokinin ribosides and *cis*-zeatin^18^.

This confirmed that CK ribosides and their monophosphates are purine ligands allegedly inducing CD via HK4^18–20^. This receptor can cooperate with HK3 one^21^, which together with HK2 are plasma- and endoplasmic reticulum (ER)-localized transmembrane proteins with extracellular and intracellular domains^22^, as evidenced in *A. thaliana*^19,20,22^, *Zea mays*^23^ and other plants^24^.

The main steps of the cytokinin transport and signalling cascade involve purine permeases (PUPs; transporters of free cytokinins), equilibrating nucleoside transporters (ENTs), subfamilies of HK-cytokinin receptors^25,26^, His phosphotransfer proteins (HPT1–5) and two types (A and B) of cytokinin gene response regulators (RRs). HPTs, small (app. 16 kDa) monomeric proteins, together with RR, initiate the cytokinin signalling cascade^26^.

A-type RRs are negative regulators of the cytokinin response, and their stabilization is dependent on phosphorylation. The B-type RRs bind to DNA and begin the expression of cytokinin-sensitive genes after their phosphorylation^26^.

It has been suggested that monophosphates interacting with HK4 or, eventually with HK3, which is present in faba beans^23^, evoke the efflux of Ca^2+^ to activate ETH-dependent CD regulators. This hypothesis is based on data showing that: (i) ETH and CKs interact at the signal transduction level^27–29^ as well as on Kin; (ii) elevated ACC levels^6^; and (iii) efflux of Ca^2+^ to the cytosol^6,9^.

To prove this hypothesis, the effects of HK ligand, regulator of CK metabolism, elements of ETH-dependent signalling pathways and amounts of cytosolic Ca^2+^ ions in Kin-PCD following chemical treatment were analysed: (i) 6-(2-hydroxy-3-methylbenzylamino purine (PI-55), a specific inhibitor of cytokinin histidine kinase receptor^30,31^; (ii) adenine (Ad), 5′-amino-5′-deoxyadenosine (Ado) and N-(2-chloro-4-piridylo)-N′-phenylurea (CPPU), inhibitors of adenine phosphoribosyl transferase (APRT)^14,32–35^, adenosine kinases (ADK)^36–38^ and CK oxidases (CKO)^39^, respectively; (iii) the effect of a plant RAF-like kinase (CTR1) inhibitor (sorafenib)^40^ on the vitality of cortex cells as well as activities of mitogen-activated protein kinase 2 (MEK2) and RAF-like kinase using Mek2^41^ and Syntide-^40,41^ substrates, respectively; (iv) ethylene glycol-bis(*β*-aminoethyl ether)-N,N,N′,N′-tetra-acetic acid (EGTA), lanthanum chloride (LaCl_3_), ruthenium red (RRed) and cyclosporine (CS-A), a chelator of extracellular Ca^2+^ ions^42^, as well as blockers of plasma-^43^, ER-^44^ membrane Ca^2+^ ion channels and MIT-PTP^45^ release Ca^2+^ ions, respectively, on the vitality of cortex cells and their cytosolic levels of Ca^2+^ ions; and (v) ETH production by seedlings treated with Ad, Ado and CPPU as well as EGTA, LaCl_3_, RRed, CS-A and sorafenib, during Kin-PCD.

Key words of the paper and List of Abbreviations used in the paper are presented in Supplementary Table S1.

## Results

**Figure 1:**
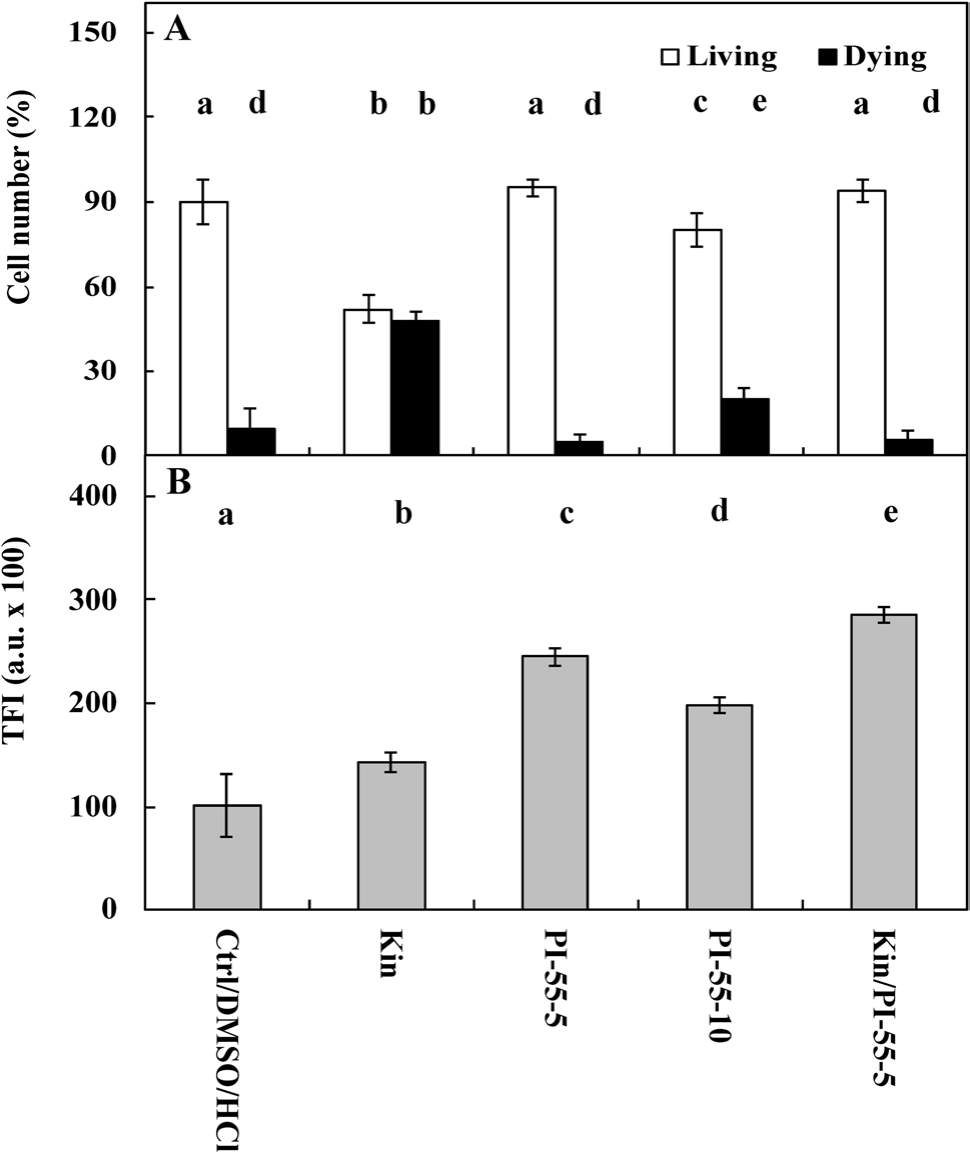
Number (%) of living and dying cells (**A**) and cytosolic calcium ion (Ca^2+^) amounts (**B**) in *Vicia faba* ssp. *minor* cortex cells of seedlings of apical part of roots after treatment with PI-55, a specific blocker of cytokinin receptors. The number of living and dying cells as well as the amounts of Ca^2+^ were fluorescently estimated after staining with acridine orange, ethidium bromide and chlortetracycline. Bars indicate ± SE of the results from three biological replicates. The means of the results with the same letter above the column are not significantly different at *p* ≤ 0.05. Ctrl, control; DMSO, dimethyl sulfoxide; Kin, kinetin; Kin/PI-55–5, kinetin with 5 μM PI-55; PI-55–5, 5 μM PI-55; PI-55–10, 10 μM PI-55.

**Figure 2:**
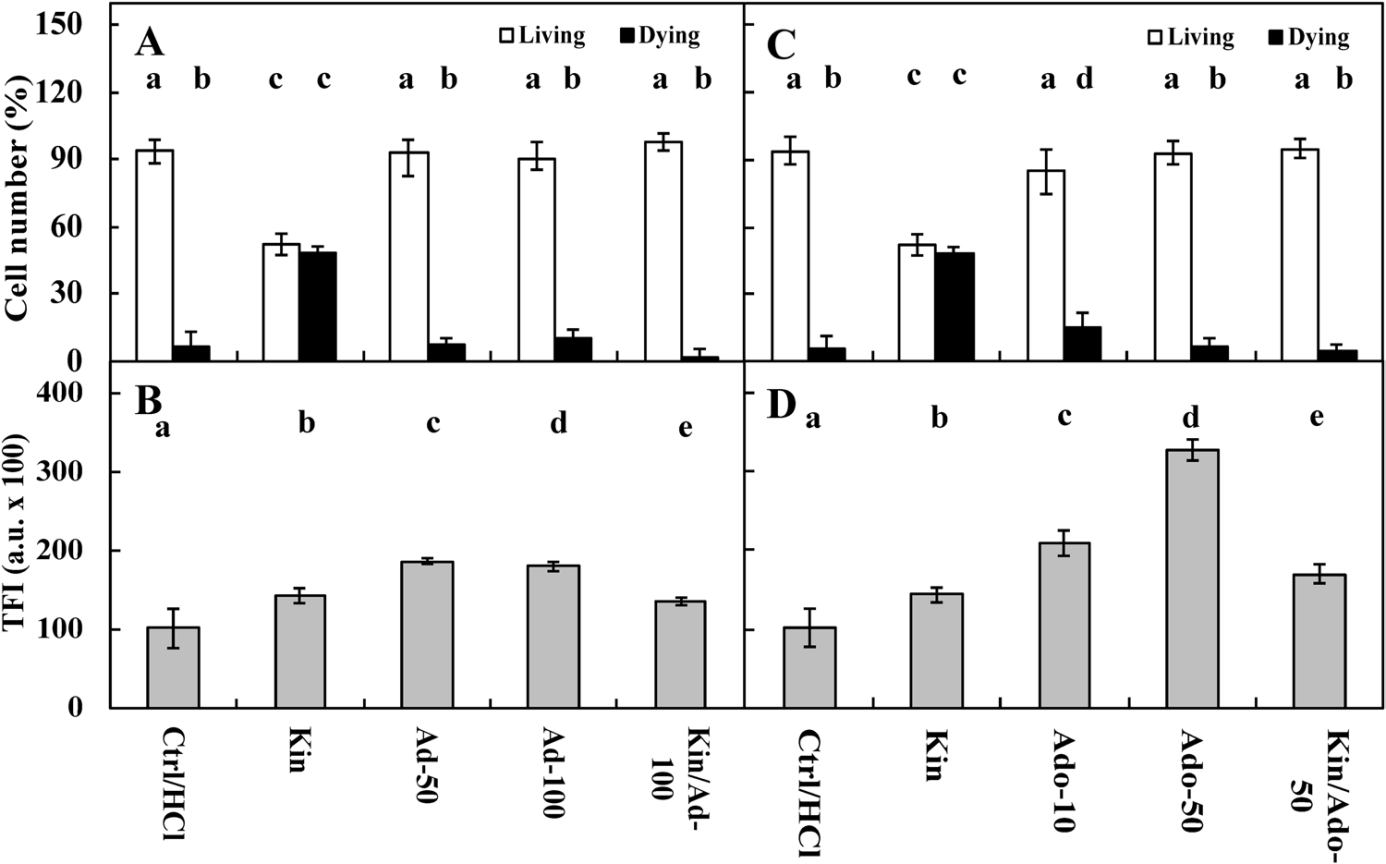
Number (%) of living and dying cells (**A**) as well as cytosolic calcium ion (Ca^2+^) amounts (**B**) in *Vicia faba* ssp. *minor* cortex seedling apical roots after treatment with Ad (adenine), an inhibitor of phosphoribosiltransferase (**A**,**B**), and Ado (5′-amine-5′-deoxyadenosine), an inhibitor of adenosine kinase activity (**C**,**D**). The number of living and dying cells as well as the amounts of Ca^2+^ were fluorescently estimated after staining with acridine orange, ethidium bromide and chlortetracycline. Bars indicate ± SE of the results from three biological replicates. The means of the results with the same letter above the column are not significantly different at *p* ≤ 0.05. Ad–50, 50 μM Ad; Ad–100, 100 μM Ad; Ado–10, 10 μM Ado; Ado–50, 50 μM Ado; Ctrl, control; Kin, kinetin; Kin/Ad–100, kinetin with 100 μM Ad; Kin/Ado–50, kinetin with 50 μM Ado; TFI, total (integrated) fluorescence intensity.

**Figure 3:**
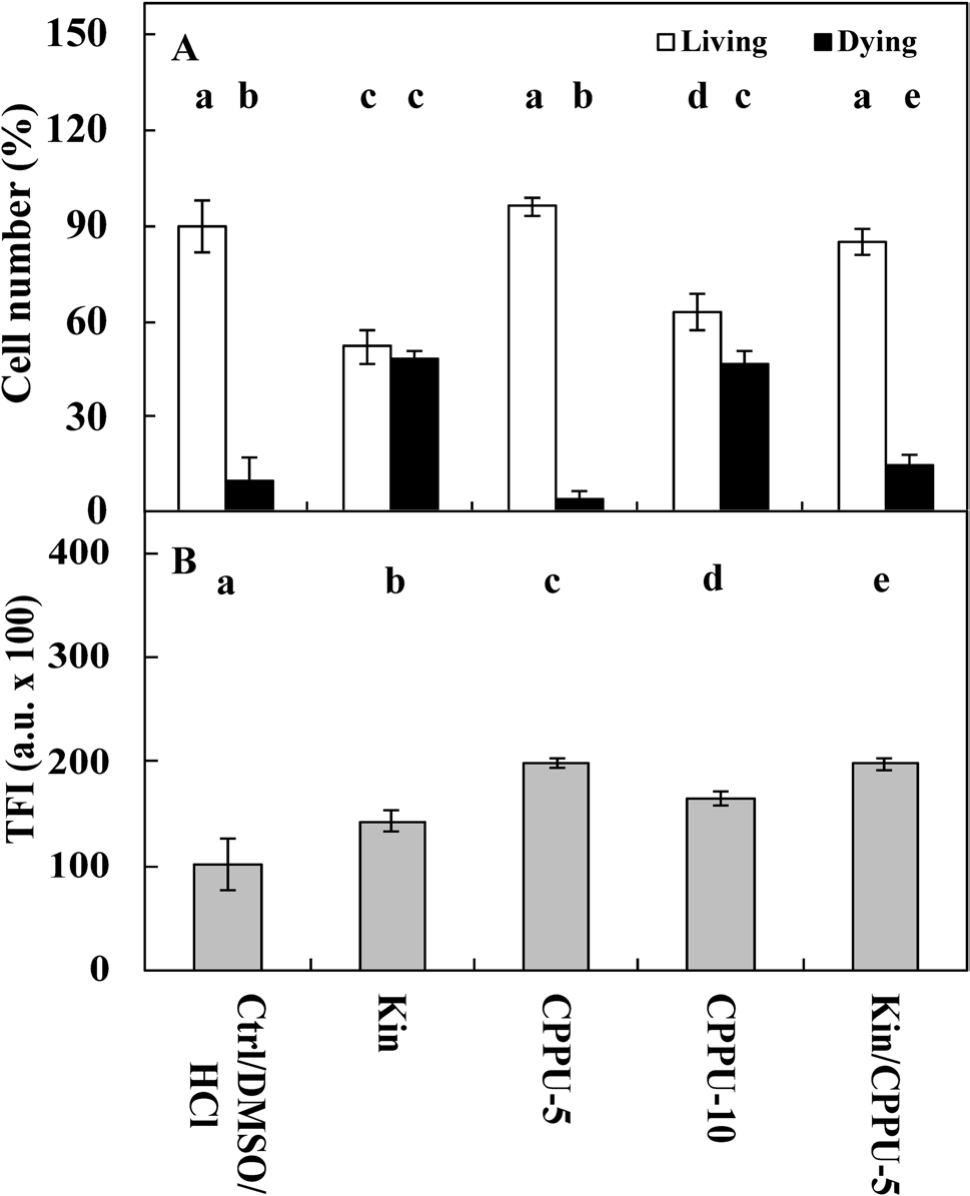
Number (%) of living and dying cells (**A**) as well as cytosolic calcium ion (Ca^2+^) amounts (**B**) in *Vicia faba* ssp. *minor* cortex seedling apical roots after treatment with CPPU, an inhibitor of cytokinin oxidases. The number of living and dying cells as well as the amounts of Ca^2+^ were fluorescently estimated after staining with acridine orange, ethidium bromide and chlortetracycline. Bars indicate ± SE of the results from three biological replicates. The means of the results with the same letter above the column are not significantly different at *p* ≤ 0.05. CPPU–5, 5 μM CPPU; CPPU–10, 10 μM CPPU; Ctrl, control; DMSO, dimethyl sulfoxide; Kin, kinetin; Kin/CPPU–5, kinetin with 5 μM CPPU.

Fluorescence intensity measurements of acridine orange (AO) and ethidium bromide (EB) in the nuclei showed that approximately 4% of Ctrl cells were dying or dead after 72 h, while the number of these cells was significantly (*p* < 0.05) greater after Kin treatment, at approximately 48% (Figs. 1A, 2A,C, 3A, 5A,C, 6A,C, 7A). The cytophotometric measurement showed that the amount of cytosolic Ca^2+^ ions in Ctrl cells was approximately 10,000 a.u., while the amount Ca^2+^ ions after Kin treatment was significantly (*p* < 0.05) greater, at approximately 14,000 a.u. (Figs. 1B, 2B,D, 3B, 5B,D, 6B,D).

### The effects of PI-55, an inhibitor of CK perception, and of Ad, Ado and CPPU, i.e., cytokinin metabolism inhibitors, on the number of living and dying cells and the amount of cytosolic Ca^2+^ ions in root cortex cells as well as on the amount of ETH in the atmosphere of growing seedlings

Five µM PI-55 (PI-55–5) did not elevate (*p* > 0.05) the CD index compared to the Ctrl series, while 10 µM (PI-55–10) did (*p* < 0.05); the number of dying cells was approximately 2% and 20%, respectively (Fig. 1A).

The amount of Ca^2+^ ions was significantly increased after treatment with 5 µM PI-55 (PI-55–5 series) (*p* < 0.05), by approximately 2.5- and 2.0-fold compared to the Ctrl and Kin series, respectively, while the amount of Ca^2+^ ions after treatment with 10 µM PI-55 (PI-55–10 series) was approximately 2.0- and 0.5-fold greater (*p* < 0.05). Thus, 5 µM PI-55 was used to study the kinetin effects on the metabolism of cortex cells. In the Kin/PI-55–5 series, the amount of Ca^2+^ ions was significantly greater (*p* < 0.05), by about 3.0- and 2.0-fold than that in the Ctrl and Kin series, respectively (Fig. 1B).

Ad, at both 50 µM and 100 µM concentrations, did not change (*p* > 0.05) the CD index compared to Ctrl cells (Fig. 2A). The amounts of Ca^2+^ ions in the Ad-50 and Ad-100 series were approximately 2.0- and 1.3-fold greater (*p* < 0.05) than those in the Ctrl and Kin series, respectively. Thus, 100 µM Ad was used to study Kin. In the Kin/Ad-100 series, the number of dying cells was similar to that in the Ctrl series (*p* > 0.05), while the amount of Ca^2+^ ions was similar to that in the Kin series (*p* > 0.05) (Fig. 2B).

Ado at 10 µM and 50 µM did not have a significant (*p* > 0.05) impact on the CD index in comparison to Ctrl series; the number of dying cells was approximately 12% and 5%, respectively. However, 50 µM Ado was used in Kin experiments. In this series, the number of dying cells was similar in Ctrl cells (Fig. 2C), while the amounts of Ca^2+^ ions in the Ado–10 and Ado–50 series were significantly greater, by approximately 2.0- and 3.0-fold, respectively, in comparison to Ctrl cells and by approximately 1.5- and 2.5-fold, respectively, in comparison with the Kin series. Meanwhile, in the Kin/Ado–50 series, the amount of Ca^2+^ ions was similar (*p* > 0.05) in the Kin series (Fig. 2D).

CPPU at 5 µM (CPPU–5 series) did not change (*p* < 0.05) the CD index compared to Ctrl cells, but after treatment with a 10 µM concentration (CPPU–10 series), the number of dying cells was similar to (*p* > 0.05) the Kin series. Thus, to study the effect on Kin-PCD, the 5 µM CPPU (Kin/CPPU–5) was used. In the Kin/CPPU–5 series, the number of dying cells was significantly lower (*p* < 0.05), by approximately 30%, than that in the Kin series (Fig. 3A). The levels of Ca^2+^ ions in the CPPU–5 and CPPU–10 series were significantly greater in comparison to Ctrl cells (*p* < 0.05), by approximately 2.0- and 1.5-fold, respectively, and by approximately 1.5- and 1.3-fold (*p* < 0.05) in comparison to Kin. Meanwhile, in the Kin/CPPU–5 series, the amount of Ca^2+^ was significantly greater in comparison to Kin (*p* < 0.05), by approximately 1.6-fold and similar to the CPPU–5 series (Fig. 3B).

**Figure 4:**
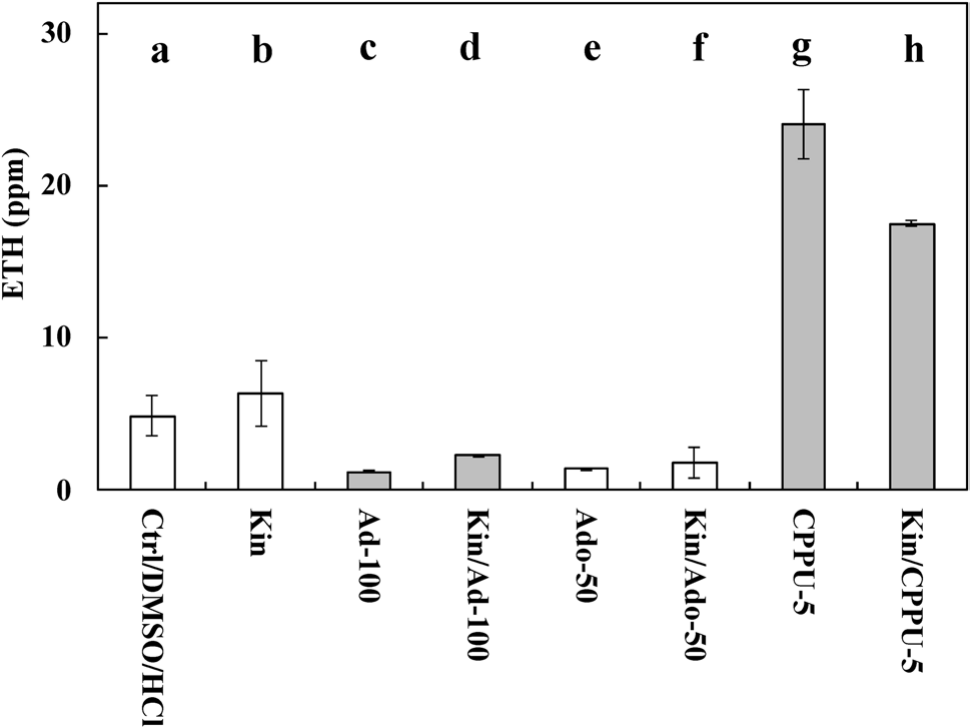
Ethylene (ETH) amounts in the atmosphere of growing *Vicia faba* ssp. *minor* of untreated (Ctrl) seedlings and those treated with Ad (adenine; 100 μM, series Ad–100), Ado (5′-amine-5′-deoxyadenosine, 50 μM, series (Ado–50), CPPU (N-(2-chloro-4-piridylo)-N′-phenylurea, 5 μM, series CPPU–5) kinetin (Kin) with 100 μM Ad (series Kin/Ad–100), or Kin with 50 μM Ado (series Kin/Ado), Kin with 5 μM CPPU (series Kin/CPPU–5). Bars indicate ± SE of the results from three biological replicates. The means of the results with the same letter above the column are not significantly different at *p* ≤ 0.05.

Measurements of ETH showed that, in the Kin series, its levels were significantly greater than in the Ctrl series (*p* < 0.05), by approximately 30% (Fig. 4).

Seedlings treated with CPPU at 5 μM (CPPU–5) produced five times (*p* < 0.05) more ETH than the Ctrl seedlings, while those treated with Kin and CPPU (Kin/CPPU–5) produced approximately three times more ETH (*p* < 0.05) than the Kin seedlings. However, its levels were 30% lower in this series than in CPPU–5 (Fig. 4).

Ad at a concentration of 100 µM or with Kin decreased the amount of ETH (Ad–100) by approximately three times (*p* < 0.05) compared to the Ctrl and Kin series, respectively (Fig. 4). Ado at 50 µM evoked similar effects to the Ad series (Fig. 4).

### Effects of EGTA and La^3+^, RRed and CS-A, chelators, and blockers of plasma-, ER-, and MIT membrane-dependent channels, respectively, on the number of dying cells induced by kinetin (Kin-PCD), the amount of cytosolic Ca^2+^ in root cortex cells and the amount of ETH in the atmosphere of growing seedlings

**Figure 5:**
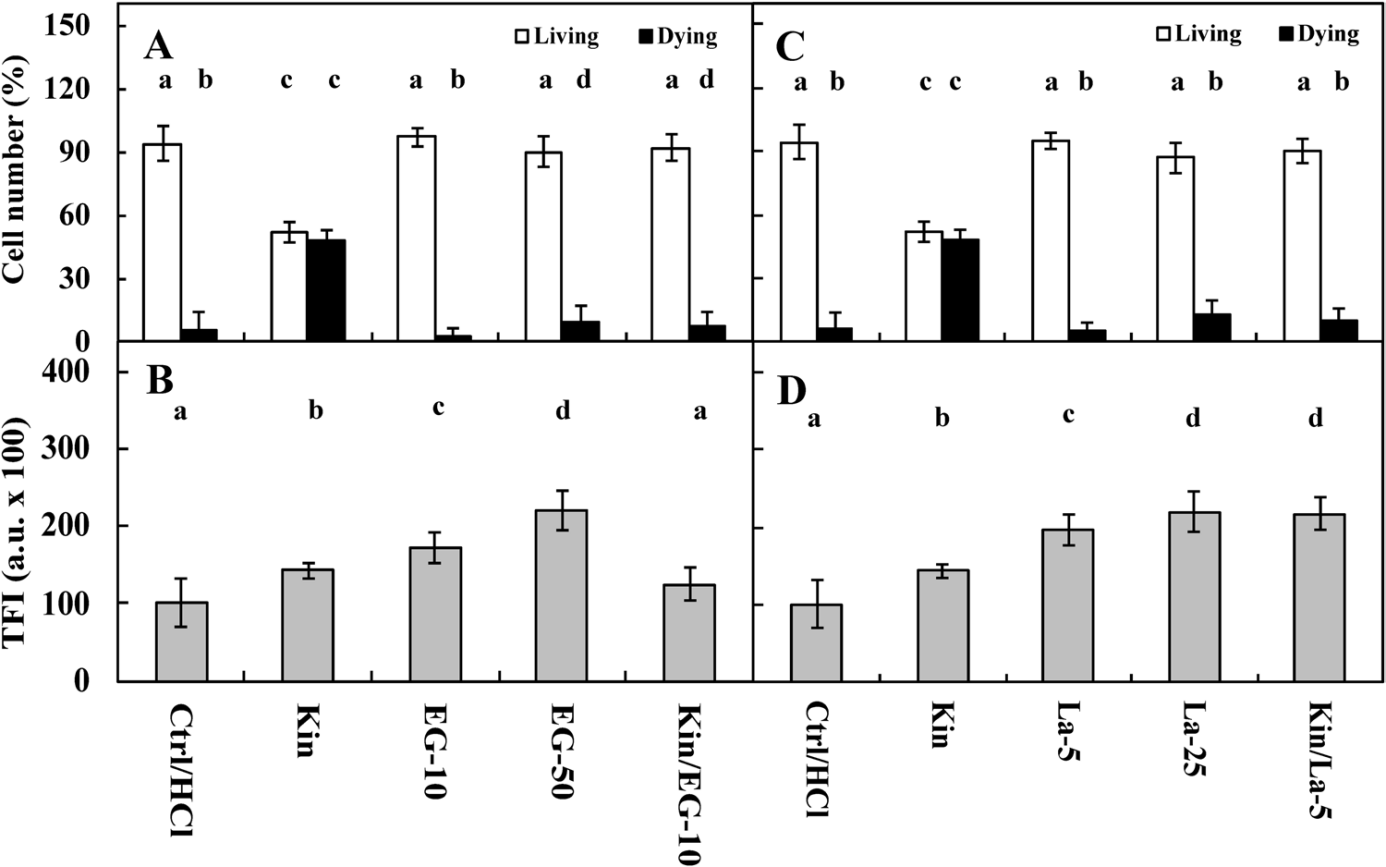
Number (%) of living and dying cells (**A**,**C**) as well as cytosolic calcium (Ca^2+^) ion amounts (**B**,**D**) in *Vicia faba* ssp. *minor* cortex seedling apical roots after treatment with EGTA (EG, ethylene glycol-bis(*β*-aminoethyl ether)-N,N,N′,N′-tetraacetic acid; **A**,**B**) and lanthanum chloride (La^3+^; **C**,**D**). The number of living and dying cells as well as the amounts of Ca^2+^ were fluorescently estimated after staining with acridine orange, ethidium bromide and chlortetracycline. Bars indicate ± SE of the results from three biological replicates. The means of the results with the same letter above the column are not significantly different at *p* ≤ 0.05. Ctrl, control; Kin, kinetin, EG–10,10 μM EGTA; EG–50, 50 μM EGTA; La–5, 5 μM La^3+^; La–25, 25 μM La^3+^; Kin/EG–10, Kin with 10 μM EGTA; Kin/La–5, Kin with 5 μM La^3+^; Kin/La–25; Kin with 25 μM La^3+^; TFI, total (integrated) fluorescence intensity.

EGTA at 10 µM (EG–10) and 50 µM (EG–50) concentrations did not change (*p* > 0.05) the number of living or dying cells compared to Ctrl cells. To study its effect on Kin-PCD, 10 µM EGTA was used (Kin/EG–10). The results showed that, in the Kin/EG–10 series, the number of dying cells was significantly lower (*p* < 0.05) than that in the Kin series, at approximately 8% (Fig. 5A). The amounts of Ca^2+^ in the EG–10 and EG–50 series were approximately 1.5- and twofold greater (*p* < 0.05) than those in the Ctrl group, respectively, and approximately 1.3- and 1.5-fold greater (*p* < 0.05) than those in the Kin group. Meanwhile, in the Kin/EG–50 group, the amount of Ca^2+^ was similar (*p* > 0.05) to that in the Ctrl group (Fig. 5B).

La^3+^ ions at 5 µM (La–5) and 25 µM (La–25) concentrations did not change the PCD index compared to Ctrl cells. To study its effect on Kin-PCD, a 5 µM concentration of La^3+^ was used. It was observed that in comparison with Kin, the number of Kin-PCD-dying cells in the Kin/La–5 series was significantly lower (*p* < 0.05), by approximately 38% (Fig. 5C). The amounts of Ca^2+^ in the La–5 and La–25 series were significantly greater (*p* < 0.05), by approximately 2- and 1.5-fold, than those in the Ctrl and Kin series, respectively. In the Kin/La–5 series, the amount of Ca^2+^ was significantly greater (*p* < 0.05), by approximately 1.5-fold, than that in the Kin series (Fig. 5D).

**Figure 6:**
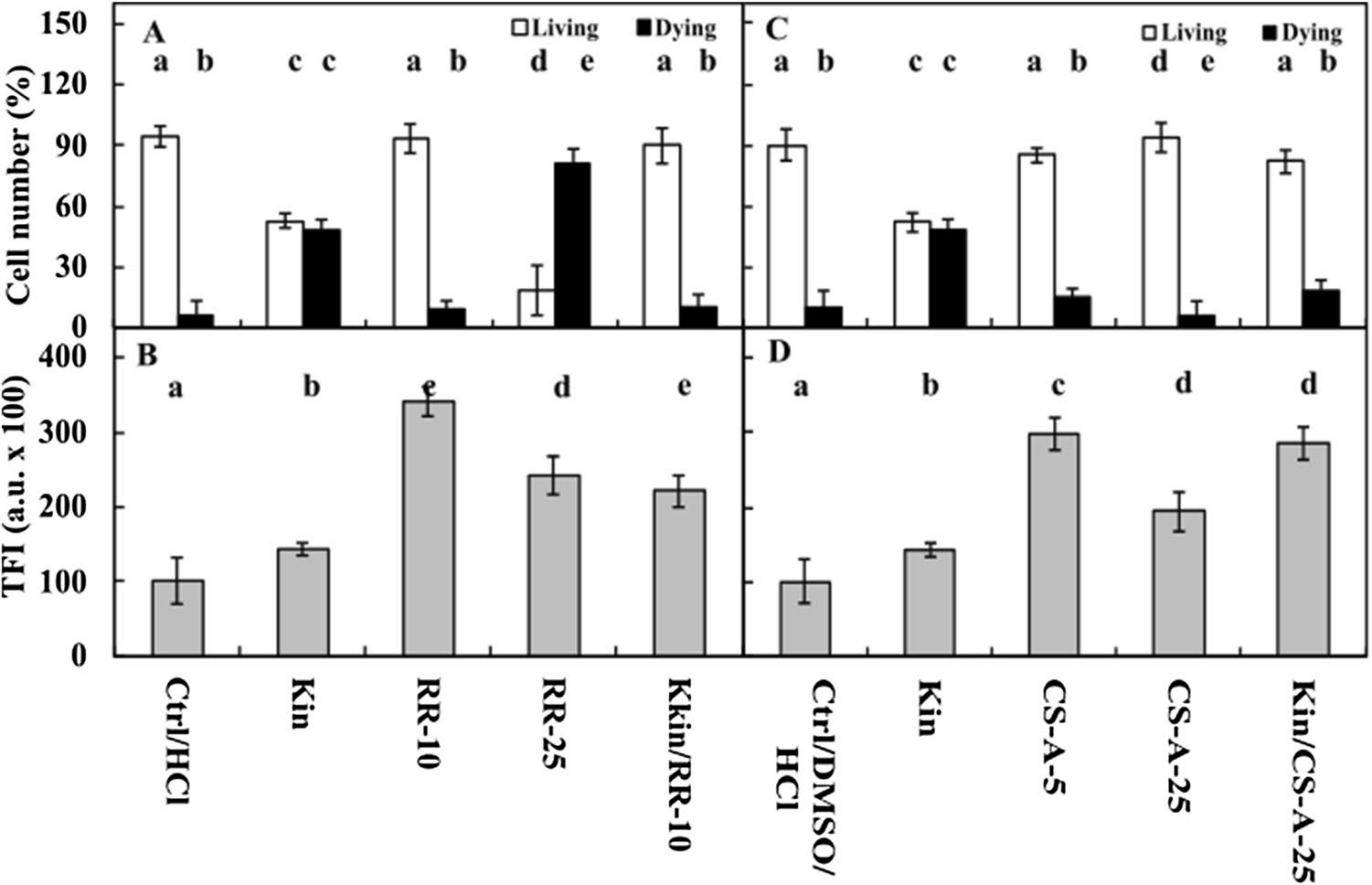
Number (%) of living and dying cells (**A**,**C**) as well as cytosolic calcium ion (Ca^2+^) amounts (**B**,**D**) in the cortex of *Vicia faba* ssp. *minor* seedling apical roots after treatment with CS-A (cyclosporine A; **A**,**B**) and RRed (ruthenium red; **C**,**D**). The number of living and dying cells as well as the amounts of Ca^2+^ were fluorescently estimated after staining with acridine orange, ethidium bromide and chlortetracycline. Bars indicate ± SE of the results from three biological replicates. The means of the results with the same letter above the column are not significantly different at *p* ≤ 0.05. Ctrl, control; DMSO, dimethyl sulfoxide; CS-A–5, 5 μM CS-A, cyclosporine A); CS-A–25, 25 μM CS-A; RRed–10, 10 μM, ruthenium red; RRed–25, 25 μM RRed; Kin, kinetin; Kin/CS-A, Kin with 25 μM CS-A; Kin/RRed–10,Kin with 10 μM RRed; TFI, total (integrated) fluorescence intensity.

RRed at a 10 µM concentration (RRed–10) did not increase (*p* > 0.05) the number of Kin-PCD-dying cells in comparison with Ctrl cells, while at a 25 µM concentration (RRed–25), it increased the number of dying cells by approximately 20- and 1.5-fold (*p* < 0.05) compared to the Ctrl and Kin series, respectively. Thus, a 10 µM concentration of RRed was used to study its effects on Kin-PCD. The results showed that 10 µM RRed inhibited Kin-induced CD; thus, the CD index was similar to that observed for the Kin series (Fig. 6A). The amounts of Ca^2+^ in the RRed–10 and RRed–25 series were significantly greater than the Ctrl and Kin series (*p* < 0.05), by approximately 3.0-fold, and by approximately 2.5- and twofold (*p* < 0.05) in comparison with Kin. In Kin/RRed–25, the amount of Ca^2+^ was approximately 2.0-fold greater than that in Kin (*p* < 0.05) (Fig. 6B).

CS-A at 5 µM concentration (CS-A–5) increased (*p* < 0.05) the number of Kin-PCD-dying cells by 2.0-fold in comparison with Ctrl cells, while it did not (*p* > 0.05) at 25 µM concentration (CS-A–25). Therefore, 25 µM CS-A (CS-A–25) was used to study its effect on Kin-PCD. In the Kin/CS-A–25 series, the number of dying cells was significantly lower compared with Kin (*p* < 0.05), by approximately 30% (Fig. 6C). The amounts of Ca^2+^ in the CS-A–5 and CS-A–25 series were significantly greater (*p* < 0.05), by approximately 3.0- and 2.0-fold, in comparison with Ctrl cells, respectively, and by approximately 2.0- and 1.5-fold greater (*p* < 0.05) in comparison with Kin. In Kin/CS-A–25, the amount of Ca^2+^ was significantly greater (*p* < 0.05), by approximately 2.0-fold, than that in Kin (Fig. 6D).

**Figure 7:**
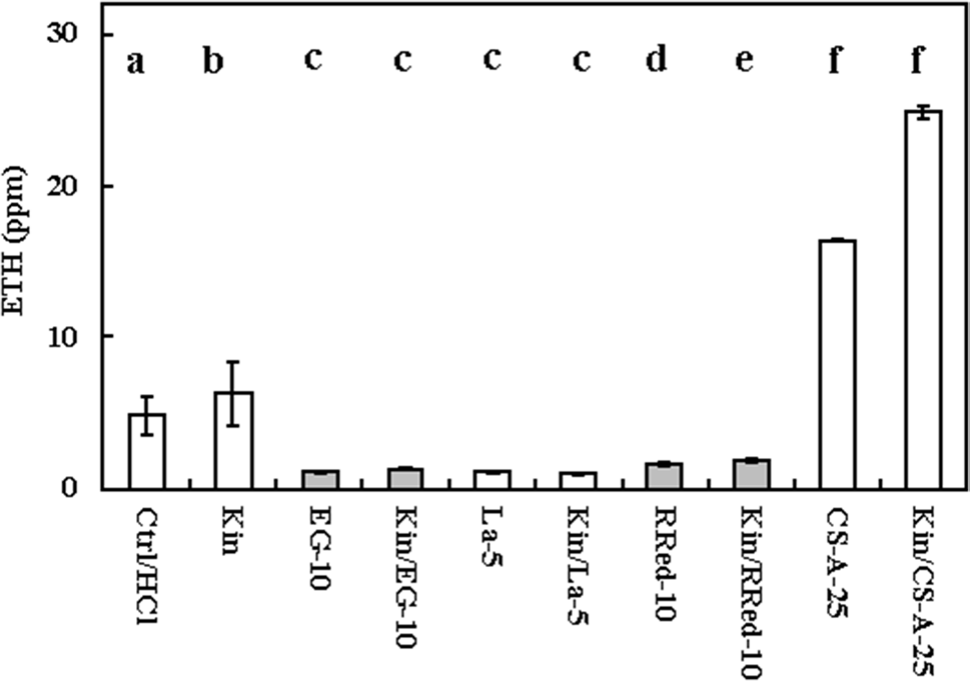
Ethylene amounts in the atmosphere of growing *Vicia faba* ssp. *minor* of control (Ctrl) seedlings and those treated with EGTA (ethylene glycol-bis(β-aminoethyl ether)-N,N,N′,N′-tetraacetic acid, 10 μM, series EG–10), La^3+^ (lanthanum, 50 μM, series La–5), (RRed (ruthenium red, 10 μM, series RRed-10) and cyclosporine A (25 μM, series CS-A–25) as well as kinetin (Kin) with EGTA (10 μM, series Kin/EG–10), Kin with La^3+^ (5 μM, series Kin/La–5), Kin with 10 μM RRed (Kin/RRed–10); Kin with 25 μM CS-A (Kin/CS-A–25). Bars indicate ± SE of the results from three biological replicates. The means of the results with the same letter above the column are not significantly different at *p* ≤ 0.05.

Measurements of ETH levels in the atmosphere of growing seedlings showed that seedlings in the EG–10, La–5 and Red–10 series had similar amounts of ETH (p > 0.05) and were approximately four times lower (*p* < 0.05) than those in the Ctrl series. In Kin/EG–10, Kin/La–5 and Kin/RRed–10, the amounts of ETH were six times lower (*p* < 0.05) than those in the Kin series. Treatment of Ctrl seedlings with CA–S at 25 μM concentration (CS-A–25) elevated the level of ETH by approximately three times (*p* < 0.05), while CS-A- and Kin-treated seedlings at the same concentration (Kin/CS-A–25) produced approximately four times greater (*p* < 0.05) levels of ETH compared to the Ctrl and Kin series (Fig. 7).

### Effect of sorafenib, an inhibitor of RAF kinase, on the number of living and dying cells during Kin-PCD and ETH as well as the activities of RAF-like and MEK2 enzymes

**Figure 8:**
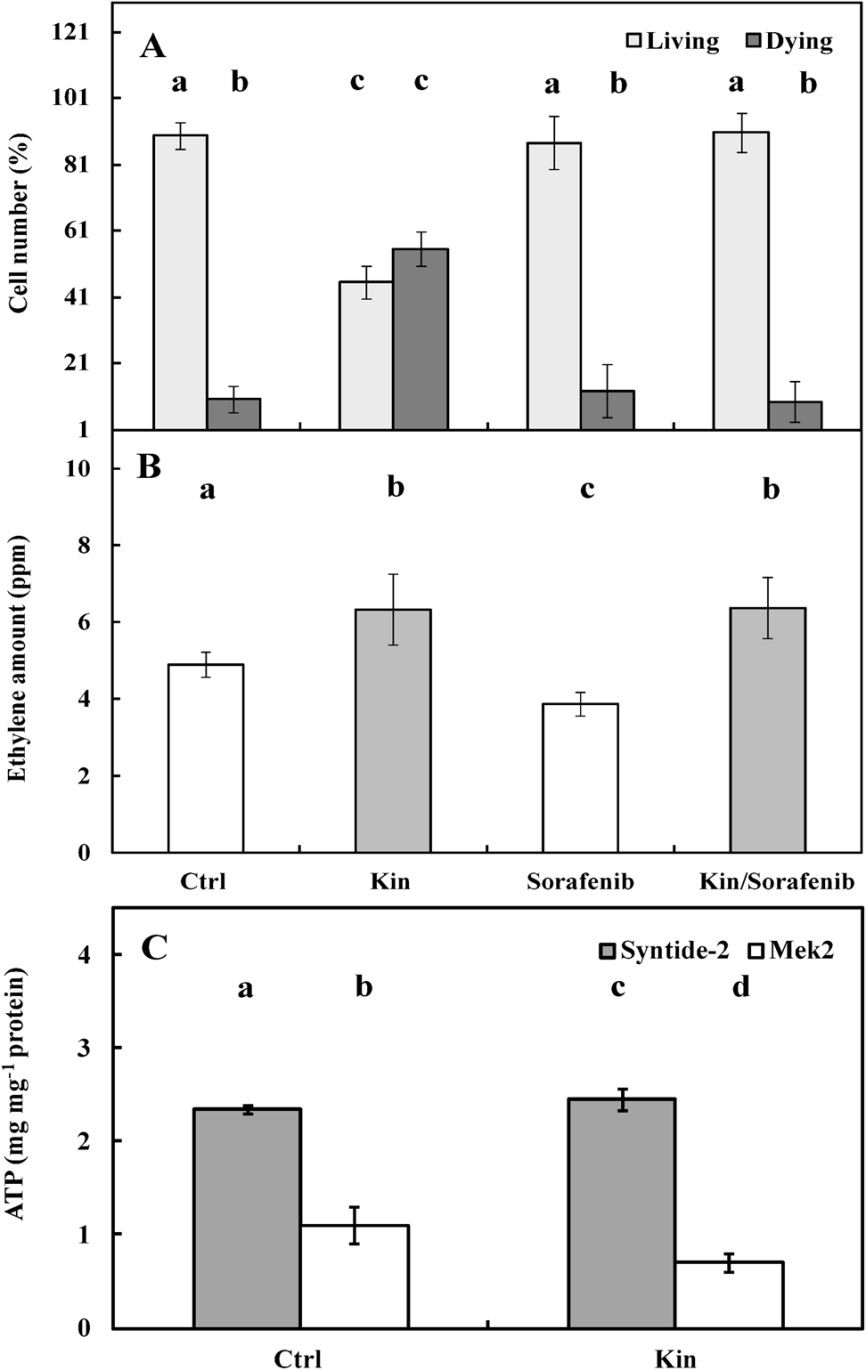
The number (%) of living and dying cells (**A**), ethylene amounts after treatment with Sorafenib (**B**) and CTR1/RAF-like and mitogen-activated protein kinase 2 (MEK2) activities with Syntide-2 and Mek2 substrate, respectively (**C**), in apical roots of *Vicia faba* ssp. *minor* seedlings treated with kinetin (Kin) for 72 h. The number of living and dying cells as well as the amounts of Ca^2+^ were fluorescently estimated after staining with acridine orange, ethidium bromide and chlortetracycline. Kinase activities were determined using the luciferase technique and ATP as a substrate. Bars indicate ± SE of the results from three biological replicates. The means of the results with the same letter above the column are not significantly different at *p* ≤ 0.05. Ctrl, control; Kin, kinetin; Sorafenib, RAF kinase inhibitor; sorafenib with Kin, Kin/Sorafenib; Syntide-2, synthetic peptide recognized as a substrate by Ca^2+^/calmodulin-dependent protein kinase II.

To estimate the number of living and dying cells, 1 µM sorafenib was used. The number of living cells in the sorafenib and Kin/Sorafenib series remained at the same level (*p* > 0.05) as in the Ctrl series. In the Kin series, the proportions of living and dying cells were approximately 45% and 55%, respectively (Fig. 8A).

In the Ctrl series, the amount of ETH per six seedlings was approximately 5 ppm, while after treatment with Kin for 72 h, its amount was approximately 30% greater (*p* < 0.05). Treatment of Ctrl seedlings with sorafenib (Sorafenib series) showed that the ETH amount was approximately 20% lower (*p* < 0.05) than that of the Ctrl seedlings. In the combination of 1 µM sorafenib with Kin (Kin/Sorafenib series), the amount of ETH was approximately 30% and 40% greater (*p* < 0.05) than that in the Ctrl and sorafenib series, respectively, and was similar (*p* > 0.05) to that in the Kin group (Fig. 8B).

Analyses of kinase activities showed that the activities of RAF-like kinase, with Syntide-2 as the substrate, and MEK2, with Mek2 as the substrate, in the Ctrl series were approximately 2.5 mg and 1 mg of ATP per mg of protein, respectively. After treatment with Kin for 72 h, their activities were approximately 20% greater (*p* < 0.05) and 20% lower (*p* < 0.05), respectively, than those of the Ctrl series (Fig. 8C).

## Discussion

Scientific reports have indicated that 10–30 μM concentrations of CKs, including Kin, fail to induce CD in either animals^16,46^ or plants^16–18,20^. In HL-60 cells, 50 μM Kin inhibited growth^47,48^, but even 100 μM Kin or BA did not induce their death^16,47,48^. In contrast, cytokinin ribosides at 10–30 μM concentrations are active cell death inducers in plants^17,18,28,47^ and animals^16,46–48^. However, death was induced by 13 μM, 27 μM and 44 μM BA as well as by 46 μM Kin of cells in *A. thaliana* plants^49^, carrots (*Daucus carota*), *A. thaliana* suspension cultures^49^ as well as cortex cells of apical parts of faba bean seedling roots^5–11^, respectively.

These facts suggested that CD depends on the organism and concentration of CKs, and a sufficient amount of free bases of CKs is important for the synthesis of their ribosides and their phosphorylated derivatives both in animal (HL-60)^16,48^ and plant cells, e.g., in *Arabidopsis* suspension cell culture^17^. There, the free zeatin becomes conjugated with ribose or ribose phosphate to produce physiologically active compounds, i.e., zeatin-9-riboside and zeatin-9-ribotide^25^.

Studies of Kin-PCD have shown that Kin in faba bean roots induces two responses^6^. The first is related to the development of protective mechanisms that act against death. Kin is an antioxidative factor that strongly inhibits oxidative and glycoxidative protein damage^5^. Such an antioxidative effect of Kin is observed in the apical parts of faba bean seedlings during Kin-PCD as increases and then decreases in ROS levels as a result of increased metabolic enzyme activities^11^. CK in Kin form can delay the senescence of detached *Raphanus sativus* leaf discs^50^. CKs inhibit leaf senescence via activation of cytokinin receptor (AHK3), the type-B response regulator (ARR2) and cytokinin response factor 6 (CRF6)^51^.

The second response^6^ of Kin in faba bean roots is related to the induction of PCD^6–11^. Kin may be degraded to Ad and ribosylated to its ribosides by APRT^14,32–35^. This enzyme was identified in wheat (*Triticum aestivum*) germs^14^. To obtain phosphorylated forms of CKs, adenosine kinase catalyses the formation of adenosine monophosphate from adenosine. Adenosine kinase was found in tobacco BY-2 cells^17^ as well as in *Arabidopsis*^27^. The next step of adenine metabolism is related to conversion of the 5′-monophosphate of zeatin to its *trans* or *cis* forms^51^. The fact that Ad and Ado, adenosine phosphorylase^14,32–35^ and adenosine kinase^36–38^ inhibitors, respectively, completely suppressed Kin-PCD in the root cortex confirmed the occurrence of such enzymes in faba bean roots and their functions.

Ad inhibits the formation of ribotides of adenine (AMP) in humans^16^ although not in *A. thaliana*. Its application to faba bean seedlings inhibited the CD induced by Kin. This fact suggested that the Ad-inhibiting activity of APRTs disrupts the CK synthesis pathway; therefore, lower amounts of CK monophosphates, which are suggested as ligands for CK receptors^16,17^, are released in the CK-dependent pathway.

Moreover, inhibition of adenosine phosphorylase and cytokinin kinase activities might enhance the inhibitory effect on Kin-PCD because an inhibitor of cytokinin oxidase, i.e., CPPU, suppressed Kin-PCD. Adenine, which is a direct product of CK degradation by cytokinin oxidase and an inhibitor of APRT activities^32–35^, acts against Kin-PCD^6^. This result indicated that the level of Kin or other CK riboside monophosphates, as a result of the activity of APRT, may be lowered. Thus, its CD-inducing effects can be decreased.

**Figure 9:**
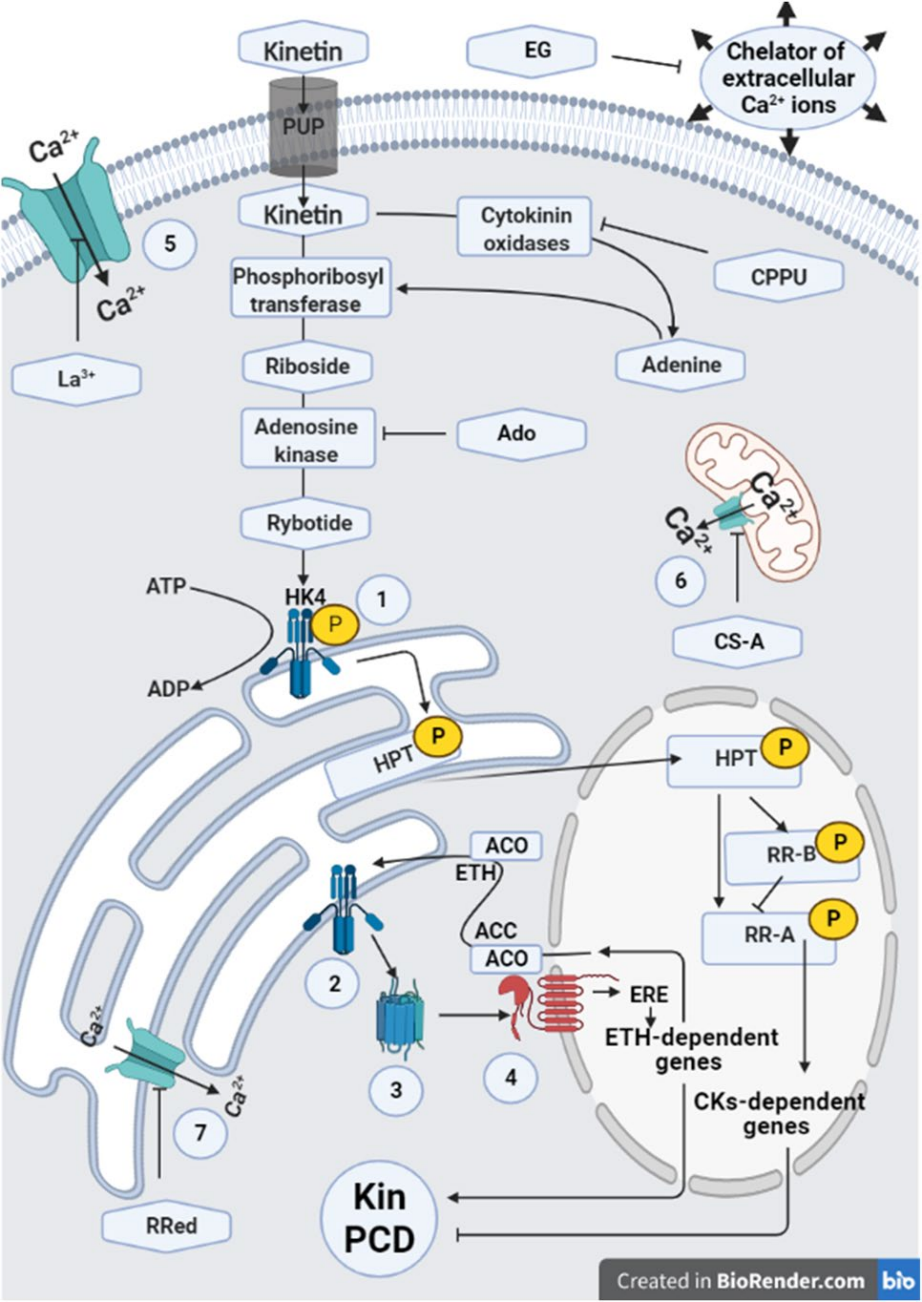
Schematic of the mechanism of the Kin-PCD signalling pathway based on the suggestions presented by Doniak et al.^6^ and Kunikowska et al.^9^ and the results described in the present study on the root cortex of *Vicia faba* ssp. *minor* seedlings. Kin transported to the cell through purine transporter (PUP) is converted to riboside and then to ribonucleotide (ribotide). The ribotide activates histidine kinase (HK) receptors (i.e., HK4), which activates the ethylene (ETH)-dependent signalling pathway using histidine-containing phosphotransmitter (HPT) and Ca^2+^-dependent channels. These channels are needed to release Ca^2+^ (also from MIT), which is important for the activation of Ca^2+^-dependent ACC (1-aminocyclopropane-1-carboxylic acid) synthase (Ca^2+-^ACS). Then, the action of serine and cysteine proteases and exo-/endonucleases of the execution/dying phase lead to protoplast and cell wall degradation, appearing as aerenchyma formation. Simultaneously, HPT may stimulate a cytokinin response regulator (RR-B) and synthesis of Ca^2+^-dependent ACC oxidase (ACO), which may enhance the PCD effect by activating ETH-dependent genes through ethylene response elements (EREs). Moreover, by stimulating reactive oxygen species (ROS) metabolism enzymes with HPT (CK-dependent genes), the protective role of Kin against Kin-PCD might be activated. (1) Cytokinin histidine kinase 4 (HK4) receptors; (2) ETH histidine kinase receptors (ETR/ERS/EIN4); (3) receptor activating constitutive triple response 1 (CTR1), a RAF-like serine/threonine (Ser/Thr) kinase; (4) ethylene insensitive (EIN2) transmembrane protein; (5) plasma membrane CNGCs (cyclic nucleotide gated channels) of calcium ions; (6) mitochondrial PTP; (7) endoplasmic reticulum (ER) membrane CNGCs; Ado, 5′-amine-5′-deoxyadenosine; CPPU, N-(2-chloro-4-piridylo)-N′-phenylurea; CS-A, cyclosporine; EG, EGTA, ethylene glycol-bis(β-aminoethyl ether)-N,N,N′,N′-tetraacetic acid; La^3+^, LaCl_3_, lanthanum chloride; RRed, ruthenium red.

Based on the results, the authors suggested that riboside monophosphates of CKs could activate faba bean HK4 receptors in endoplasmic reticulum (ER) membranes^18,19^. HK4 has a greater affinity for monophosphorylated forms of cytokinin ribosides and free cytokinin bases than HK319; however, both receptors can interact with each other^22^. This might result from the fact that PI-55 completely inhibited the Kin-induced death of cortex cells. It is known that dimerization and autophosphorylation of HKs lead to the translocation of the phosphate group from histidine to aspartic acid, the regulatory domain of the receptor. Then, histidine HPT carriers and type B RR, discovered in *A. thaliana*^26^*,* whose homologues may exist in faba bean, can activate the cytokinin-dependent response (Fig. 9).

During Kin-PCD, a decrease in the levels of ATP^7^, activities of cellular dehydrogenases^9^ and histone kinase activities^7^ is observed, suggesting that ATP is used for the synthesis of CK riboside monophosphates and/or cytotoxic N^6^-furfuryladenosine (Kin-riboside), as in human cancer cell lines^52^.

The idea that Kin-PCD depends on the cooperation of Kin with ETH comes from the results of studies with aminooxyacetic acid (AOA), CoCl_2_ and 2,5-norbornadiene (NBD), inhibitors of the synthesis and conversion of ACC to ETH as well as its receptor, respectively, which suppress Kin-PCD^6^, and from results showing that Kin elevated the levels of ACC^9^ and ETH (present paper). Loss of HK4 function in the *ahk4* mutant decreased the ETH response to CKs, indicating that the *A. thaliana* HK4 receptor is probably a primary contributor to ETH biosynthesis in response to CKs^29,53^. Therefore, HPT of *A. thaliana* can transduce signals from CKs^29,53,54^ (Fig. 9) to ETH receptors (ethylene triple response, ETR1 and 2; ethylene insensitive 4, EIN4; ethylene response sensor 1 and 2 ERS1 and 2 in ER membranes^29,53^. Moreover, CKs can posttranscriptionally increase the activity of *ACS 4,5* gene products^55^, leading to an increase in the activities of these enzymes in ETH synthesis. Thus, it can synergistically increase the ETH amount in faba plants (Figs. 4, 7). ETH can activate EIN2- and ER- and nuclear ETH-dependent membrane receptors and induce ETH response elements (EREs)^29,53,56^. The fact that aerenchyma was formed after Kin-^6^ and ACC-induced^13^ death of cortex cells of faba bean root confirmed the hypothesis because ETH seemed to be a direct hormonal factor involved in the process^42^. This was also confirmed by the results presented in the paper showing that Ad, Ado and CPPU, inhibitors of CK synthesis enzymes, i.e., APRT, ADK and CKO, respectively, reduced the Kin-induced death of cells, but the level of ETH only increased in the CPPU series.

CPPU could participate in the alternative ETH-CK pathway, which was postulated by Kin-PCD authors. The results showed that sorafenib, an inhibitor of ATP-competitive kinase^40^, suppressed Kin-PCD, which was manifested by a low number of dying cells, similar to the Ctrl series. However, the RAF-like CTR1 kinase activity, the mitogen-activated protein kinases (MAP kinases), a structural equivalent of serine/threonine-specific protein kinase (RAF kinase), element of the MAP-kinase-dependent cascade, the regulator of ETH signal transduction pathway^57^, was greater in roots of Kin than in Ctrl series, while the MEK2 activity was lower compared to Ctrl series. The differences in the activities of these kinases may have resulted from the fact that sorafenib can also affect the activity of other kinases, e.g., ERK (extracellular signal-regulated kinase)^40^. These facts allow us to suppose that both kinases exist in faba bean roots.

However, the results indicated that a decrease in the number of dying cells depended on the effect of an increase in RAF-like kinase activity and a decrease in MEK2 activity. Moreover, the results confirmed that Kin-PCD is the process controlled by cooperation among ETH-dependent MAP kinase signalling pathways. The crosstalk between Kin and ETH might also take place at the level of CK and ETH receptors (the two-component systems)^25–28,54^ because both receptors are members of the HK family proteins^18–20,22,29,53^.

Results presented by Scharein and Groth^27^ also confirmed the existence of ETH-CK crosstalk. The phosphorylation between ETR1 and histidine-containing phosphotransfer protein 1 (AHP1) complexes plays a crucial role in this process. The affinity between the two partners is greater when one of them is phosphorylated and the other is not^27^.

The present results also showed that the amounts of cytosolic Ca^2+^ in the root cortex cells in faba bean seedlings in PI-55–5 and CPPU–5 were similar to Kin/PI-55–5 and Kin/CPPU–5, but they were greater in Ad–100 and Ado–50 in comparison with Kin/Ad–100 and Kin/Ado–50. The levels of cytosolic Ca^2+^ in Kin/PI-55–5 and Kin/CPPU–5 were greater than in Kin, while the levels were similar between the Kin/Ad–100 and Kin/Ado–50 and Kin series.

The application of EGTA, La^3+^, RRed and CS-A, i.e., chelators of extracellular Ca^2+^ ions^43^, inhibitors of plasma-^43^, in a nitrate^3^ or chloride form, ER-^44^ and MIT-^45^ membrane Ca^2+^ ion channels, respectively, inhibited the Kin-PCD process. Additionally, the levels of cytosolic Ca^2+^ in cortex cells in Kin/La-5, Kin/RRed-10 and Kin/CS-A-25 were greater in comparison with Ctrl and in Kin series (Figs. 5, 6), but the amounts of Ca^2+^ in Kin/EG-10 series were similar to the Ctrl (Fig. 5).

The idea that Ca^2+^ ions are important for Kin-PCD induction was suggested previously^7^. The results of the paper clearly indicated that inhibition of Kin-PCD takes place when plasma-, ER-membrane Ca^2+^ ion channels and MIT-PTP cyclophilin dependent on release of Ca^2+^ ions are blocked or extracellular sources of Ca^2+^ are not available, leading to limitations of the migration and maintenance of noninductive cell death levels of these ions (Fig. 9). Therefore, blocked MIT permeability pores protected against the release of ROS and the swelling of MIT, some of the most important factors^58,59^ related to the hallmark^58,59^ of cell death. Both phenomena were observed during Kin-PCD^6,11^.

Doniak et al*.*^3^ and Kunikowska et al*.*^6^ suggested an alternative role of Ca^2+^ in the transduction of Kin-dependent signals. A greater amount of Ca^2+^ ions was also important for the activation of Kin-PCD via calcium-dependent ACC synthase (ACS) and ACC oxidase (ACO), enhancing^55,60^ ACC and ETH synthesis, respectively. It is also possible that ETH initiated the expression of protein kinases and/or their activities, especially H1, and core histones^7^ induced ETH-positive feedback on their own synthesis via the same enzymes (Fig. 9).

The existence of one or more alternative pathways inducing and transducing Kin-PCD was confirmed by the results showing that the elevated levels of ETH during Kin-PCD were lowered by two CK metabolism inhibitors (AD and Ado), EGTA, a chelator of extracellular Ca^2+^ ions, and by two Ca^2+^ ion channel blockers, i.e., La^3+^ and RRed. In this process, CPPU and CS-A elevated the Kin-PCD-dependent ETH levels.

The other alternative pathway of Kin-PCD in relation to Ca^2+^ ions and ETH may depend on phosphatidylinositol 4,5-bisphosphate and inositol 1,4,5-trisphosphate^4^ because the phosphoinositide cycle is required for the regulation of ACS activity^61^.

The participation of ETH in Kin-PCD can also be confirmed by the fact that this process is accompanied by an ETH-dependent triple response^3,6,10^, i.e., shortening and thickening of roots as the effect changes in the same way as cortex cell expansion and root apical hook formation^3,6^, as well as by aerenchyma formation in Kin-PCD^3,6^. Thus, aerenchyma formation should be added as the next feature of ETH-dependent but not triple or quadruple responses.

## Conclusions

The results based on the usage of reagents, such as PI-55, Ad, Ado, CPPU, EGTA, La^3+^_,_ RRed and CS-A, sorafenib, Syntide-2 and Mek2, the inhibitor of RAF kinase as well as RAF-like and MEK2 substrate related to the animals, plants and bacteria were crucial for explaining Kin-PCD progression.

First and foremost is the fact that Kin controls cell differentiation in both plants and animals. Second, CTR1 kinase, known in animals and bacteria as RAF kinase, forms transmembrane receptors for CKs, and ETH also exists in plants as RAF-like receptors.

Taking all the results into account, we can conclude that the Kin-dependent signalling pathways by the phosphorylated forms of Kin or other CK ribosides may activate CD-dependent HK4 (strongly suggested as the cell death receptor activated by CKs) receptors and generate pathways via HPT, enhancing the expression of *ACS4,5* genes and the activities of Ca^2+^-dependent ACSs. Then, by activation of Ca^2+^-dependent ACOs, the ACC and ETH amounts increase (Fig. 9).

Then, the signal is transferred onto specific ETH-dependent genes, and it induces: (i) expression of elements initiating the executive phase of Kin-PCD, including serine and caspase-like proteolytic and nucleolytic machinery; and (ii) cell wall compound metabolism, which finally leads to complete degradation of some cells in the degradation phase of Kin-PCD, resulting in aerenchyma formation.

We demonstrated that perception and metabolism of CK inhibitors completely suppressed Kin-PCD. However, PI-55, Ado and CPPU increased amounts of cytosolic Ca^2+^, but Ad did not reduce the Kin-induced changes. Ad and Ado decreased while CPPU increased the Kin-elevated amounts of ETH.

Moreover, the chelator of extracellular Ca^2+^ ions as well as plasma-, ER- and MIT-membrane Ca^2+^ channel inhibitors also suppressed Kin-PCD. However, except for EGTA, they increased the Kin-elevated amounts of cytosolic Ca^2+^ in the cortex cells of seedling roots. EGTA, La^3+^ and RRed decreased and CS-A increased the amount of ETH.

Thus, Ca^2+^ ions are the central link of cooperation between ETH and Kin in the studied Kin-PCD.

## Materials and methods

### Plant material, chemicals, and experiments

*V. faba* ssp. *minor* cv. Bobas (Danko, Sobiejuchy 2, 88–400 Żnin, Sobiejuchy 2, Poland; http://www.danko.pl) seeds (20) were germinated for 3 days in Petri dishes (15 cm in diameter and 3 cm high) on two blotting papers with distilled water (the seeds were half submerged) in a dark breeding room. For analyses, 6 of the 3-d-old seedlings with nearly equal root length (2.0 ± 0.3 cm) were transferred into a glass container (8 cm in diameter and 4 cm high) with two blotting papers moistened with 10 cm^3^ of water (Ctrl) or adequate solutions of chemicals and cultivated at 23 ± 1 °C and 92% ± 2% relative humidity for exactly 72 h and then used for analyses. The types, sources and solvents of the factors originally used in the studies are presented in Table 1.

**Table 1:** The concentrations, solvents, experimental destinations and origin of the modulators of cytokinin reception and metabolisms as well as blockers of calcium ions channels and its release from mitochondria as well as inhibitor of Raf-like kinase activities and RAF and MEK2 kinase substrates applied in the studies.

	Factor—concentration—solvent	Destination		Origin
1.	6-(2-hydroxy-3-methylbenzylamino purine (PI-55)—5 and 10 µM—0.05% dimethylsulfoxide (DMSO)	Inhibitor of cytokinin receptors	Laboratory of Growth Regulators, Palacký University	
2.	Kinetin (Kin)—46 µM—0.01 N HCl water solution	Inducer of cell death in root cortex cells	Sigma-Aldrich Company	
3.	Adenine (Ad)—50 and 100 µM—water	Inhibitor of adenine phosphoribosyl transferase (APRT)	Sigma-Aldrich Company	
4.	5′-amino-5′-deoxyadenosine (Ado)—10 and 50 µM—water	Inhibitor adenosine kinases (ADK)	Sigma-Aldrich Company	
5.	N-(2-chloro-4-piridylo)-N′-phenylurea (CPPU)—5 and 10 µM—0.1% methanol	Inhibitors CK oxidases	Sigma-Aldrich Company	
6.	Cyclosporine (CsA)—5 and 25 µM—0.1% methanol	Blocker of calcium ions release by permeability transition pore (PTP)	Sigma-Aldrich Company	
7.	Ethylene glycol-bis(β-aminoethyl ether)-N,N,N′,N′-tetra-acetic acid (EGTA)—10 and 50 µM—water	Blocker of all membrane calcium channels	Sigma-Aldrich Company	
8.	Lanthanum chloride (LaCl_3_)—5 and 25 µM—water	Blocker of endoplasmic reticulum membrane calcium channels	Sigma-Aldrich Company	
9.	Sorafenib—1 µM—1% DMSO water solution	RAF kinase inhibitor	Selleckchem	
10.	Mek2—1 µg per 1 ml—1% DMSO water solution	MEK2 kinase substrate	Selleckchem	
11.	Syntide-2—1 µg per 1 ml—1% DMSO	RAF kinase substrate	Selleckchem	

First, the impact of CK perception and metabolism regulators as well as Ca^2+^ channel inactivators at two selected concentrations (Table 1) without Kin on CD and cytosolic Ca^2+^ amounts in the faba bean seedling root cortex was tested. Then, the concentration of the factor that did not induce or slightly induced cell death compared to the other was used to analyse its respective influence on the vitality and amount of cytosolic Ca^2+^ in cortex cells during Kin-induced CD.

### Quantification of cell viability

Analyses of cell viability were performed in the cortex of untreated plants (Ctrl) and those treated with kinetin (46 μM; series Kin), PI-55 (5 μM and 10 μM, series PI-55–5 and PI-55–10), Ad (50 μM and 100 μM, series Ad–50 and Ad–100), Ado (10 μM and 50 μM, series Ado–10 and Ado–50), CPPU (5 μM and 10 μM, series CPPU–5 and CPPU–10), EGTA (10 μM and 50 μM, series EG–10 and EG–50), La^3+^ (5 μM and 25 μM; series La–5 and La–25), RRed (10 μM and 25 μM, series RRed–10 and RRed–25) and CS-A (5 μM and 25 μM; series CS-A–5 and CS-A–25) as well as with Kin (46  μM) and 5 μM PI-55 (series Kin/Pi-55–5), Kin and 100 μM Ad (series Kin/Ad–100), Kin and 50 μM Ado (series Kin/Ado–50), Kin and 5 μM CPPU (series Kin/CPPU–5), Kin and 10 μM EGTA (series Kin/EG–10), Kin and 5 μM La^3+^ (series Kin/La–5), Kin and 10 μM RRed (series Kin/RRed–10) and with Kin and 25 μM CS-A (series Kin/CS-A–25).

The viability (Supplementary Fig. S1) of cells was also analysed after treatment with sorafenib, a RAF-like kinase inhibitor, at 1.0 µM concentration in cells treated and untreated with Kin *V. faba* ssp. *minor* seedlings (Supplementary Fig. S2).

Additionally, the effects of solvents used for PI-55, CS-A, and Kin as well as for CPPU preparation, i.e., 0.05% DMSO (Avantor), 0.01 N HCl (Avantor) and 0.1% methanol and a mixture of HCl and DMSO as well as HCl with methanol (Avantor) in distilled water, respectively, on the viability of cortex cells were tested, and the average values were used to complete the figures. Moreover, the effects of the mentioned solvents on the fluorescence intensity of CTC/cytosolic Ca^2+^ complexes used to determine the relative amounts of Ca^2+^ were assessed, and the results of studies referred to the respective Ctrl values.

To detect and measure the intensity of CD (percentage of dying cells) in the root cortex, apical fragments of roots were cut off from the seedlings, washed twice with 0.1 M PHB (Na phosphate buffer pH 7.4; Avantor), stained with a mixture of 100 μg cm^−3^ acridine orange (AO; Sigma–Aldrich) and 100 μg cm^−3^ ethidium bromide (EB; Sigma–Aldrich) in PHB, washed in PHB two times and fixed with a 2% solution of glutaraldehyde in Na-phosphate buffer (PHB). Then, thin sections of the long axes of 2-cm apical parts of roots were analysed and photographed under blue light from an Optiphot-2 fluorescence filter (B2A) (Nikon, Japan) equipped with a DXM1200 digital camera and Act-1 (Precoptic, Poland) software.

Estimation of the numbers of living, dying and dead cortex cells was carried out according to the method described in Doniak et al*.*^6^, Byczkowska et al.^13^ and Kunikowska et al*.*^9^ using the specially prepared calibration curve, which presents relationships between the fluorescence intensity (FI) of dyes and the amount of nuclear chromatin. Measurements of FI were carried out using Scion Image (Scion Corporation) software. During measurements, each stained nucleus was separately outlined using the threshold function, and the values of fluorescence intensity in arbitrary units were then read and compared to the calibration curve of Byczkowska et al.^13^.

This method uses the properties of EB-migration, in which the amount in nuclei increases proportionally with the CD-induced increase in plasma and nuclear membrane permeabilization. AO migration through all types of membranes does not depend on their conditions. Thus, the changing colour of nuclear chromatin ranging from green to orange-red is related to increasing fluorescence intensity (FI; Supplementary Fig. 3S; A)^13^.

Bright-orange (Fig. S3; A, a1) and orange–red (Fig. S3; A, a2) colours indicate dead cells (FI values > 46 a.u.), yellow (Fig. S3; A,a3) and yellow–orange (Fig. S3; A,a4) indicate dying ones (FI values 34–55 a.u.), while green–yellow (Fig. S3; A,a2) and green indicate living cells, with resultant fluorescence intensity (RFI) values of 10–35 a.u. (Fig. S3; A,a1).

FI values of viability were reported as indices. The data represent the means ± SE of two replicates of three independent experiments (n = 3) from approximately 450–550 cells.

### Estimation of cytosolic Ca^2+^ ion content

Analyses of cytosolic Ca^2+^ amounts were made in the cortex of untreated plants (Ctrl) and those treated with Kin (46 μM), PI-55 (5 μM and 10 μM, series PI-55–5 and PI-55–10), Ad (50 μM and 100 μM, series Ad-50 and Ad-100), Ado (10 μM and 50 μM, series Ado–10 and Ado–50), CPPU (5 μM and 10 μM, series CPPU–5 and CPPU–10), EGTA (10 μM and 50 μM, series EG–10 and EG–50), La^3+^ (5 μM and 25 μM, series La–5 and La–25), RRed (10 μM and 25 μM, series RRed–10 and RRed–25), CS-A (5 μM and 25 μM, series CS-A–5 and CS-A–25) as well as with Kin and 5 μM PI-55 (series Kin/Pi-55–5), Kin and 100 μM Ad (series Kin/Ad–100), Kin and 50 μM Ado (series Kin/Ado–50), Kin and 5 μM CPPU (series Kin/CPPU–5), Kin and 10 μM EGTA (series Kin/EG–10), Kin and 5 μM La^3+^ (series Kin/La–5), Kin and 10 μM RRed (series Kin/RRed–10) and with Kin and 25 μM CS-A (series Kin/CS-A–25).

To measure the cytosolic Ca^2+^ amount in cortex cells, 2-cm-long apical parts of faba bean roots (between the 4th and 20th mm from the apex) were fixed with a 2% solution of glutaraldehyde (POCH) in PHB for 1 h and stained with 100 μM chlortetracycline (CTC; Merck-Sigma), and longitudinal handmade (approximately 300–400 μm thick) sections were prepared. Then, analyses were carried out under a B2A filter of an epifluorescence microscope (Fig. S3; B, b1–b6), and the total green fluorescence intensity (TFI) of Ca^2+^-CTC complexes was cytophotometrically measured using Scn Image software using photos^6,9^.

During measurements, each stained cell was separately outlined using the threshold option, and then the values of fluorescence intensity in a.u. were read and used to calculate the relative Ca^2+^ amounts. The decreasing amount of Ca^2+^ was related to the values of green TFI of Ca^2+^-CTC complexes (Fig. S3; B,b1–b6). The data represent the means ± SE of two replicates of three independent experiments (n = 3) from approximately 500–600 cells.

### Estimation of ETH amount, RAF-like kinase and MEK2 activities and protein amount

ETH measurements were carried out in Erlenmeyer flasks before sample preparations. Erlenmeyer flasks were sealed with aluminium foil (to keep seedlings in the dark) and tightly closed with caps with clogged pipette tips. After 30 min of incubation, a handheld ETH analyser (SCS56, Storage Control System, United Kingdom) equipped with a pump was used. It was connected to pipette tips via a flexible tube directly before measurement (Fig. 2S). Then, the pump was turned on, and measurements were conducted for 30 s. The results were read between 20 and 30 s, when the values reached the plateau; the five readings from the monitor were written in a spreadsheet of MS Excel and taken to calculate the ETH amount in ppm per six seedlings.

ETH was measured in Ctrl and Kin series and after treatment with 100 μM Ad (series Ad–100), 50 μM Ado (series Ado–50), 5 μM CPPU (series CPPU–5), 10 μM EGTA (series EG–10), 5 μM La^3+^ (series La–5), 10 μM RRed (series RRed–10) and 25 μM CS-A (series CS-A–25) and with 1 μM sorafenib (series Sorafenib) as well as after treatment with kinetin (46 μM, Kin) and with above mentioned factors, i.e. with Kin and 100 μM Ad (series Kin/Ad–100), Kin and 50 μM Ado (series Kin/Ado–50), Kin and 5 μM CPPU (series Kin/CPPU–5), Kin and 10 μM EGTA (series Kin/EG–10), Kin and 5 μM La^3+^ (series Kin/La–5), Kin and 10 μM RRed–10 (series Kin/RRed–10) and Kin and 25 μM CS-A (series Kin/CS-A–25) and Kin and 1 μM sorafenib (series Kin/Sorafenib).

To estimate RAF-like and MEK2 kinase activities, one-third of the length of apical parts of roots was homogenized and reextracted in 0.04 M Tris–HCl pH 7.5 buffer (Sigma–Aldrich) containing 20 mM MgCl_2_, 10 µg ml^–1^ BSA (bovine serum albumin; Sigma–Aldrich) and 1 mM PMSF (phenylmethylsulfonyl fluoride; Sigma–Aldrich) in 1.5 ml Eppendorf-like tubes with a plastic mortar and centrifuged at 5000*g* for 10 min^7^.

The reaction mixture for kinase activity analyses was prepared by sequentially adding 2-ml tubes: the extract (20 µl), extraction buffer (1035 µl), ATP (5 µl), Syntide-2 or Mek2 (25 µl; 1 µg protein per 1 ml of buffer) as substrates and Kinase-Glo Reagent (50 µl; Promega), containing luciferase and luciferin. After mixing, the luminescent signals were measured in a semimicrofluorometer cell with a Teflon Stopper by a fluorescence/luminescence spectrophotometer F – 2500 (Hitachi) at 458 nm wavelength every for 2.5 min for 30-s intervals. The kinase activities were calculated as the difference between the luminescence of the samples without substrates and the luminescence of the amount of ATP not utilized by kinase substrate phosphorylation. The kinase activity was expressed in relative light units (RLU), indicating the RLU amount of ATP utilized by kinases in 1 mg of protein.

To estimate the protein amounts, the apical parts of roots were homogenized with 100 mM Tris–HCl (pH 7.4) buffer in Eppendorf-like tubes using a plastic pestle (4–8 °C) and then centrifuged at 5000×*g* for 10 min (4 °C); residues were then re-extracted. Combined supernatants were used to measure protein amounts in the reaction mixture containing 2-ml Eppendorf-like tubes, extract (10–100 µl), extraction buffer (90–0 µl) and Coomassie Brilliant Blue G-250 reagent^56^ (1.4 ml). Absorbance was measured at 595 nm after 10 min of incubation (Amersham Biosciences Ultrospec 1100 Pro UV–VIS spectrophotometer with semi-micro cell).

To calculate the protein amount, a standard, i.e., BSA dissolved in PHB, was prepared in a range of 5 to 100 μg of protein in a 100 µl volume, and measurements were carried out according to the above description. The prepared calibration curve was used to read the amount of protein in the sample and final calculations.

Protein estimation reagent was prepared with Coomassie Brilliant Blue G-250 (100 mg) diluted in 95% ethanol (50 ml), 85% H3PO4 (100 ml) and 950 ml of distilled water and stored in a dark bottle.

### Statistics and software

Three biological replicates, at least in tri-, duplicate and more random samples, were analysed. The samples were prepared from at least six plants. The results of measurements were statistically verified by the Mann–Whitney U test and/or Student’s t-test using MS EXCEL software (licenced) by independent step-by-step analyses of each column of results. Significant differences between results were observed at p ≤ 0.05. Calculations, all charts, and tables were prepared using MS EXCEL software (licenced).

To estimate the vitality of cells, by counting the number of live, dying, and dead cells, an Optiphot-2 epifluorescence microscope (Nikon) with a blue filter (B2A) equipped with a digital camera (DXM1200) and objectives (10×, 20×, 40×) ACT-1 software (Precoptic, Poland) and SCION IMAGE (Scion Corporation) software (open source) were used.

CORELDRAW GRAPHICS SITE X7 EDULIC and/or INSCAPE (open source) were used to prepare figures and image planes in tiff extensions. Images of seedlings were taken using a Canon 100 (Japan) camera in jpg extension.

BIORENDER software was used to prepare Fig. 9.

### Confirmation

The authors confirmed that permission of usage of seeds of V. faba spp. minor var. ‘Bobas’ for scientific application in the Department of Cytophysiology, Pomorska 141/143, 90–236 Łódź, Poland was obtained by Danko Hodowla Nasion Sp. z o.o.

### Declaration

The authors confirm that all experiments with seedlings of nongenetically modified *Vicia faba* ssp. *minor* were performed in accordance with relevant guidelines and regulations using operation instructions for the laboratory equipment and measuring instruments. All applied study methods were performed in accordance with the relevant guidelines and regulations using protection equipment against hazards. Moreover, all images have been taken by the authors of the manuscript of the paper.

## Supplementary Information

Supplementary Information.

## Data Availability

The datasets generated and analysed during the current study are available from the corresponding author on reasonable request.
