# Supplementary material for: Mechanism of kinetin-induced death of Vicia faba ssp. minor root cortex cells
Source: Sci Rep. 2021 Dec 9;11:23746. doi: 10.1038/s41598-021-03103-3 (PMC8660813; doi:10.1038/s41598-021-03103-3)
Supplement: Supplementary file 1 — Supplementary Information. [file 41598_2021_3103_MOESM1_ESM.docx]

**Mechanism of kinetin-induced death of *Vicia faba* ssp. *minor* root cortex cells**

Andrzej Kaźmierczak^1✉^, Anita Kunikowska^1^, Magdalena Doniak^1^ & Andrzej Kornaś^2^

^1^Andrzej Kaźmierczak

^1^University of Łódź, Faculty of Biology and Environmental Protection, Institute of Experimental Biology, Department of Cytophysiology, Pomorska 141/143, 90-236 Łódź, Poland

^1^Anita Kunikowska

^1^E-mail: anita.kunikowska@ec1lodz.pl

https://orcid.org/0000-0002-0459-9351

^1^Magdalena Doniak (M.D.)

^1^E-mail: [mdoniak@lokolo.onmicrosoft.com](mailto:mdoniak@lokolo.onmicrosoft.com)

<https://orcid.org/0000-0002-4550-8452>

^2^Andrzej Kornaś

^2^Institute of Biology, Pedagogical University of Krakow, Podchorążych 2, 30-084 Kraków, Poland

andrzej.kornas@up.krakow.pl

https://orcid.org/0000-0001-9945-3322

(^✉^ Corresponding author)

^1^E-mail: andrzej.kazmierczak@biol.uni.lodz.pl

Fax: +48 +42 635-45-12

Tel: +48 +42 635-42-03

http://orcid.org/0000-0001-6446-7337

PBN: 3936870

**Supplementary Figures**

**Figure S1.** Untreated (Ctrl; **A**) and kinetin- (Kin; **B**), Sorafenib- (RAF kinase inhibitor; **C**) as well as Kin and Sorafenib-treated (**D**) for 3 days *Vicia faba* ssp. *minor* seedlings. Images were taken using the Canon 100 camera in jpg extension. **a** - seed; **b** - stem; **c** - root, **d** - root hairs, **e** - apical hook. Scale bars = 20 mm.

**Figure S2.** Images of nuclei of cortex cells of *Vicia faba* ssp. *minor* seedling roots stained with acridine orange and ethidium bromide after Sorafenib treatment. Ctrl (**A,A’**), Kin- (**B,B’**), Sorafenib- (**C,C’**) and Kin with Sorafenib- (**D,D’**) treated seedlings. **a** - nuclei of alive cells; **b**,**d** - nuclei of dying cells. Scale bars in A,A’ = 20 µm, are applied to all figures.

**Figure S3.** Graph (**A**) and panels with the green (**a6**), green-yellow (**a5**), yellow (**a4**), yellow-orange (**a3**), bright-orange (**a2**) and orange-red (**a1**) colour and nuclei respectively, used to calculate the number of living (**a6,a5**; fluorescence intensity values (FI) values 10–35 a.u.), dying (**a4,a3**; FI values 34–55 a.u) and dead (**a2,a1**) cells after acridine orange and ethidium bromide staining as well as graph (**B**) with the dark to green (**a6-a1**; FI values 0 and 400 a.u.) panels used to estimate calcium ion (Ca^2+^) amount. Scale bars in A1 and B1 = 10 µm, are applied to all figures. **TFI** – total (integrated) fluorescence intensity.

.

**Supplementary Table 1**

| **List** | Description |
| --- | --- |
| K**eywords** | calcium ions; cytokinin receptors; calcium ion channels, cytokinin and ethylene signal transduction pathway; ethylene, fluorescence; histidine kinases; pore transition pore; programmed cell death. |
| **Abbreviations** | ACC – 1-aminocyclopropane-1-carboxylic acid; ACS – Ca^2+^-dependent ACC synthase; Ad – adenine; ADK – adenosine kinases; Ado – 5'-amine-5'-deoxyadenosine; AHP-1 – histidine-containing phosphotransfer protein 1; AO – acridine orange; AOA – aminooxyacetic acid; APRT – adenine phosphoribosyl transferase; BA – benzyl adenine; EB – ethidium bromide; CD – cell death; CDKs – cyclin dependent kinases; CHK – cytokinin receptor; CoCl_2_ – cobalt chloride; CK/CKs – cytokinin/cytokinins; CKO – cytokinin oxidases; CNGC – cyclic nucleotide gated channels; CPPU – N-(2-chloro-4-piridylo)-N’-phenylurea; CS-A – cyclosporine A; CTC – chlortetracycline; CTR1 – serine/threonine-protein kinase, RAF-like kinase; DMSO – dimethylsulfoxide; dPCD – developmental PCD; ePCD – environmental PCD; EB – ethidium bromide; EGTA – ethylene glycol-bis(*β*-aminoethyl ether)-N,N,N',N'-tetraacetic acid; EIN – ethylene receptor (ethylene insensitive); ENT – nucleoside transporters; ER – endoplasmic reticulum; ERE – ethylene response element; ERK – extracellular signal-regulated kinase; ERS – ethylene receptor, i.e., ethylene response sensor; ETH – ethylene; ETR – ethylene receptor, i.e., ethylene triple response; FI – fluorescence intensity; HK – histidine kinase; HPT – His-containing phosphotransmitter; Kin – kinetin; KR – kinetin riboside; KRMP – kinetin riboside monophosphate; LaCl_3_ – lanthanum chloride; MAP kinase – mitogen-activated protein kinase; MEK2 – mitogen-activated protein kinase 2; MG115 – Z-Leu-Leu-Nva-H; NBD – 2,5-norbornadiene; MIT – mitochondrion; NBD – 2,5-norbornadiene; NEM – N-ethylmaleimide; PCD ‒ programmed cell death; PHB – Na-phosphate buffer; PI-55 – 6-(2-hydroxy-3-methylbenzylamino)purine; PMSF – phenylmethylsulfonyl fluoride; PTP – permeability transition pore; ­­PUP – purine permease; RAF kinase – serine/threonine-specific protein kinase; RFI – resultant fluorescence intensity; RAF kinase – serine/threonine-specific protein kinase; ROS – reactive oxygen species; RR – cytokinin gene response regulator; RRed – ruthenium red; SOD – superoxide dismutase; TFI – total (integrated) fluorescence intensity. |

**Table S1.** List of Key words and Abbreviations used in the paper.
